# Methylglyoxal modulates endothelial nitric oxide synthase-associated functions in EA.hy926 endothelial cells

**DOI:** 10.1186/1475-2840-12-134

**Published:** 2013-09-19

**Authors:** Yang Su, Syed M Qadri, Lingyun Wu, Lixin Liu

**Affiliations:** 1 Department of Pharmacology, College of Medicine, University of Saskatchewan, 107 Wiggins Road, Saskatoon, SK, Canada; 2 Department of Health Sciences, Lakehead University and Thunder Bay Regional Research Institute, Thunder Bay, ON, Canada

**Keywords:** Methylglyoxal, eNOS uncoupling, Superoxide, Tyrosine nitration, Biopterins, eNOS phosphorylation

## Abstract

**Background:**

Increased levels of the sugar metabolite methylglyoxal (MG) *in vivo* were shown to participate in the pathophysiology of vascular complications in diabetes. Alterations of endothelial nitric oxide synthase (eNOS) activity by hypophosphorylation of the enzyme and enhanced monomerization are found in the diabetic milieu, and the regulation of this still remains undefined. Using various pharmacological approaches, we elucidate putative mechanisms by which MG modulates eNOS-associated functions of MG-stimulated superoxide $\left(O_{2}^{•-}\right)$ production, phosphorylation status and eNOS uncoupling in EA.hy926 human endothelial cells.

**Methods:**

In cultured EA.hy926 endothelial cells, the effects of MG treatment, tetrahydrobiopterin (BH4; 100 μM) and sepiapterin (20 μM) supplementation, NOS inhibition by N^G^-nitro-L-arginine methyl ester (L-NAME; 50 μM), and inhibition of peroxynitrite (ONOO^-^) formation (300 μM Tempol plus 50 μM L-NAME) on eNOS dimer/monomer ratios, Ser-1177 eNOS phosphorylation and 3-nitrotyrosine (3NT) abundance were quantified using immunoblotting. $O_{2}^{•-}–$dependent fluorescence was determined using a commercially available kit and tissue biopterin levels were measured by fluorometric HPLC analysis.

**Results:**

In EA.hy926 cells, MG treatment significantly enhanced $O_{2}^{•-}$ generation and 3NT expression and reduced Ser-1177 eNOS phosphorylation, eNOS dimer/monomer ratio and cellular biopterin levels indicative of eNOS uncoupling. These effects were significantly mitigated by administration of BH4, sepiapterin and suppression of ONOO^-^ formation. L-NAME treatment significantly blunted eNOS-derived $O_{2}^{•-}$ generation but did not modify eNOS phosphorylation or monomerization.

**Conclusion:**

MG triggers eNOS uncoupling and hypophosphorylation in EA.hy926 endothelial cells associated with $O_{2}^{•-}$ generation and biopterin depletion. The observed effects of the glycolysis metabolite MG presumably account, at least in part, for endothelial dysfunction in diabetes.

## Introduction

Chronic hyperglycemia fosters endothelial dysfunction that accounts for the pathophysiology of microvascular sequelae in diabetes [1,2]. The elevated glycolysis metabolite methylglyoxal (MG) has been implicated in vascular complications such as hypertension [3], impaired microcirculation [4] and thrombosis [5] in diabetes. The increased MG affects multi-organ homeostasis by modulating immune cell functions [6], cytokine induction [7], cytosolic Ca^2+^[8], cellular energy and redox balance [9] and adhesion molecules expression [10] and induces both necrotic [11] and apoptotic cell death [4].

Endothelial nitric oxide synthase (eNOS) is the predominant and constitutively expressed NOS in vascular endothelial cells and catalyzes the reaction for generation of nitric oxide (NO) from L-arginine in the presence of the cofactors tetrahydrobiopterin (BH4) and NADPH [12]. Regulation of eNOS activity is coupled to cytosolic Ca^2+^[13] whereas the expression is regulated by a wide range of transcriptional and posttranscriptional mechanisms [14]. Alterations of NO balance contribute to the pathophysiology of diabetic complications [15]. Deficiency of either L-arginine or BH4 results in reduced NO but enhanced superoxide $\left(O_{2}^{•-}\right)$ production, a functional change of eNOS that is defined as eNOS uncoupling [12]. eNOS uncoupling is associated with increased eNOS monomerization, tyrosine nitration and formation of dihydrobiopterin (BH2) and decreased cellular BH4 [16,17]. Sepiapterin is a stable precursor of BH4 and serves as a valuable pharmacological agent for the study of eNOS uncoupling due to its high cell permeability as compared to BH4 [18,19]. $O_{2}^{•-}$ avidly reacts with NO to form peroxynitrite (ONOO^-^) which triggers the oxidation of BH4, impairs eNOS activity and induces eNOS uncoupling [12,16,20].

Uncoupling of eNOS is the underlying mechanism of endothelial dysfunction associated with cardiovascular conditions such as hypertension, stroke, and heart failure [21,22]. Recently, eNOS uncoupling was shown to participate in endothelial dysfunction in diabetic mice [23] and to mediate peripheral neuropathy in diabetic rats [24]. In Zucker diabetic fatty rats, endothelial dysfunction and decreased NO availability were attributed to dissociation of eNOS from HSP90, an effect elicited by increased calpain activity [25]. As a myriad of molecules are dysregulated in diabetes, the specific effects of MG on eNOS uncoupling, however, remain elusive.

Ramifications of elevated MG levels in hyperglycemia include impaired NO production and redox imbalance [26]. Various studies promulgate a possible link between endothelial dysfunction and functional alterations of eNOS after MG treatment. To date, however, discrepant data prevail on MG sensitivity of eNOS functions in different model systems. On the one hand, MG was shown to stimulate transcription of eNOS [27]; while on the other, abundance of eNOS protein was reduced following MG treatment [28,29]. In contrast, MG was shown to suppress eNOS phosphorylation on serine-1179 without affecting eNOS protein expression [30]. Increased MG levels in preeclamptic vasculature were shown to be coupled with enhanced arginase, LOX-1 and tyrosine nitration [31]. The association between MG-triggered eNOS phosphorylation, eNOS uncoupling, and oxidative stress in vascular endothelial dysfunction, however, remains ill-defined.

The present study explores the mechanisms of MG-induced endothelial dysfunction by examining putative eNOS-associated functions. We elucidate the effects of exogenous BH4 and sepiapterin administration, NOS inhibition and suppression of peroxynitrite (ONOO^-^) formation on $O_{2}^{•-}$ generation, eNOS monomerization, cellular biopterin levels, tyrosine nitration, and phosphorylation of eNOS in EA.hy926 endothelial cells *in vitro*.

## Materials and methods

### Cell culture and pharmacological treatments

EA.hy926 cells, the hybrid human umbilical vein endothelial cell line cells [32], were obtained from American Type Culture Collection (Rockville, MD, USA) and cultured in Dulbecco’s modified Eagle’s medium (Cellgro, VA, USA) with 10% fetal bovine serum (Hyclone, UT, USA), 100 U/mL penicillin and 100 μg/mL streptomycin (Amresco, OH, USA) with 5% CO_2_ and maximal humidity at 37°C. Cells between passage 3 and 6 were used for the experiments. As indicated in the figure legends, various pharmacological approaches were used to elucidate eNOS-associated functions. To maintain eNOS dimerization, the NOS cofactor 5,6,7,8-tetrahydrobiopterin (BH4, 100 μM; Sigma-Aldrich, Oakville, ON, Canada) or the negative control, a pteridine analogue, 5,6,7,8-tetrahydroneopterin (NH4, 100 μM; Schircks Laboratories, Jona, Switzerland) were freshly-prepared and administered. NH4 has similar antioxidant effects as BH4 but, unlike BH4, is ineffective in restoring uncoupled eNOS [33]. Sepiapterin (20 μM; Cayman, Ann Arbor, MI, USA), a substrate for BH4 synthesis via the pterin salvage pathway, was used to increase cellular BH4 levels [34]. The NOS inhibitor N^G^-nitro-L-arginine methyl ester (L-NAME, 50 μM; Sigma-Aldrich), was administered to inhibit uncoupled eNOS-derived $O_{2}^{•-}$[35]. For suppression of ONOO^-^ generation, EA.hy926 cells were treated with a combination of the ROS scavenger 1-oxyl-2,2,6,6-tetramethyl-4-hydroxypiperidine (Tempol, 300 μM; Santa Cruz, Dallas, TX, USA) and NOS inhibitor L-NAME (50 μM) for 24 h prior to the addition of MG [36,37]. $O_{2}^{•-}$ reacts with NO at equimolar ratio to generate ONOO^-^[36], and from our unpublished observations, pretreatment with a combination of L-NAME and Tempol more potently inhibits ONOO^-^ formation than pretreatment with either pharmacological agent alone.

### Determination of $O_{2}^{•-}$ production

$O_{2}^{•-}$ levels were determined using a commercial superoxide detection kit (Enzo, Brockville, ON, Canada) according to the manufacturer’s instructions as described previously [38,39]. The superoxide detection reagent when oxidized produces an orange fluorescent compound which is retained in the cell. Cells were seeded in a 96-well plate and stained with the membrane-permeable and nonfluorescent $O_{2}^{•-}$ detection probe (5 μM, 30 min, 37°C in the dark). Excessive probe was removed by washing with PBS. The fluorescence in cells was detected using excitation and emission wavelengths of 544 nm and 590 nm respectively. The data are expressed as arbitrary units.

### Analysis of biopterin levels by HPLC

Measurement of BH4 and total biopterins was performed by fluorometric HPLC analysis as described previously with slight modifications [35]. After indicated treatments, EA.hy926 cells were lysed in a lysis buffer (pH 7.4; containing 50 mM Tris–HCl, 1 mM DTT and 1 mM EDTA) with 0.1 μM neopterin (Sigma-Aldrich) as an internal recovery standard. The samples were deproteinated with 10% 1:1 mixture of 1.5 M HClO_4_ and 2 M H_3_PO_4_ and centrifuged (12,000 × g for 10 min). The supernatant was split into portions and subjected to acid- and alkali-oxidation respectively. For determination of total biopterins (BH4, BH2 and non-reduced biopterins) by acid-oxidation, 10 μl iodine solution (1% iodine in 2% KI solution) was added into each 90 μl supernatant and for quantification of BH2 and non-reduced biopterins by alkali-oxidation, 10 μl of 1 M NaOH and 10 μl of iodine solution were added to 80 μl supernatant. Following incubation (1 h, room temperature in the dark), the alkali-oxidation samples were acidified with 1 M H_3_PO_4_, the iodine in both acid- and alkali-oxidation samples was reduced using 5% ascorbic acid (Sigma) and the samples were centrifuged (12,000 × g for 10 min). The supernatant was collected for HPLC analysis using a Hitachi D-7000 HPLC system (Hitachi, Mississauga, ON, Canada) via Symmetry C18 reverse-phase column with a methanol–water (1.5:98.5, v/v) mobile phase running at 0.5 mL/min. Fluorescence was detected at excitation and emission wavelengths of 348 nm and 444 nm respectively. The level of BH4 was calculated by subtracting BH2 and non-reduced biopterins from total biopterins and expressed as pmol per mg protein. The biopterin levels in NH4-treated cells were calculated without adding 0.1 μM neopterin.

### Western blotting

Non-reducing SDS-PAGE was performed to detect eNOS dimers and monomers [35]. Briefly, cultured EA.hy926 cells were harvested and lysed on ice for 30 min by using RIPA buffer (50 mM Tris–HCl, 150 mM NaCl, 1% NP-40, 0.5% sodium deoxycholate, 0.1% SDS and protease inhibitors cocktail, pH 8.0). The lysate was centrifuged at 10,000 × g for 10 min, and the supernatant was collected, incubated with 4 × Laemmli buffer without β-mercaptoethanol (200 mM Tris–HCl, 50% glycerol, 2% SDS, 0.04% bromophenol blue, pH 6.8) at 37°C for 5 min. Aliquots of cell lysates (50 μg of protein each) were separated on 7% SDS-PAGE. Gels and buffers were equilibrated at 4°C before electrophoresis, and the buffer tank was placed in an ice bath during electrophoresis to maintain the low temperature. Subsequent to non-reducing SDS-PAGE, the gels were electrotransferred to a nitrocellulose membrane (Bio-Rad, CA, USA), and the blots were probed by primary antibody against eNOS (1:1000; BD Pharmingen, USA) and horseradish peroxidase-conjugated secondary antibody (Santa Cruz) as routine Western blot. In separate gels, total eNOS (1:1000), β-actin (1:1000; Santa Cruz, CA, USA), phospho-eNOS (Ser-1177, 1:1000; BD Pharmingen, CA, USA) and 3-nitrotyrosine (3NT, 1:1000; Enzo) from cell lysates of the same experiments were detected by routine Western blot under reduced condition. The bands were visualized with enhanced chemiluminescence reagents (GE Healthcare Life Sciences, NJ, USA) and exposed to X-ray film (Kodak scientific imaging film, ON, Canada). Densitometric quantification of the detected bands was performed using Quantity One® Software (Bio-rad). The ratio of eNOS dimer to monomer was normalized to one in the absence of MG.

### Statistical analysis

Data are expressed as arithmetic means ± SEM from at least three independent experiments. Statistical analysis was made using one way analysis of variance (ANOVA) with Tukey’s post-hoc comparison test. n denotes the number of different batches of cells tested in each treatment group. Values of p < 0.05 were considered statistically significant.

## Results

**Figure 1:**
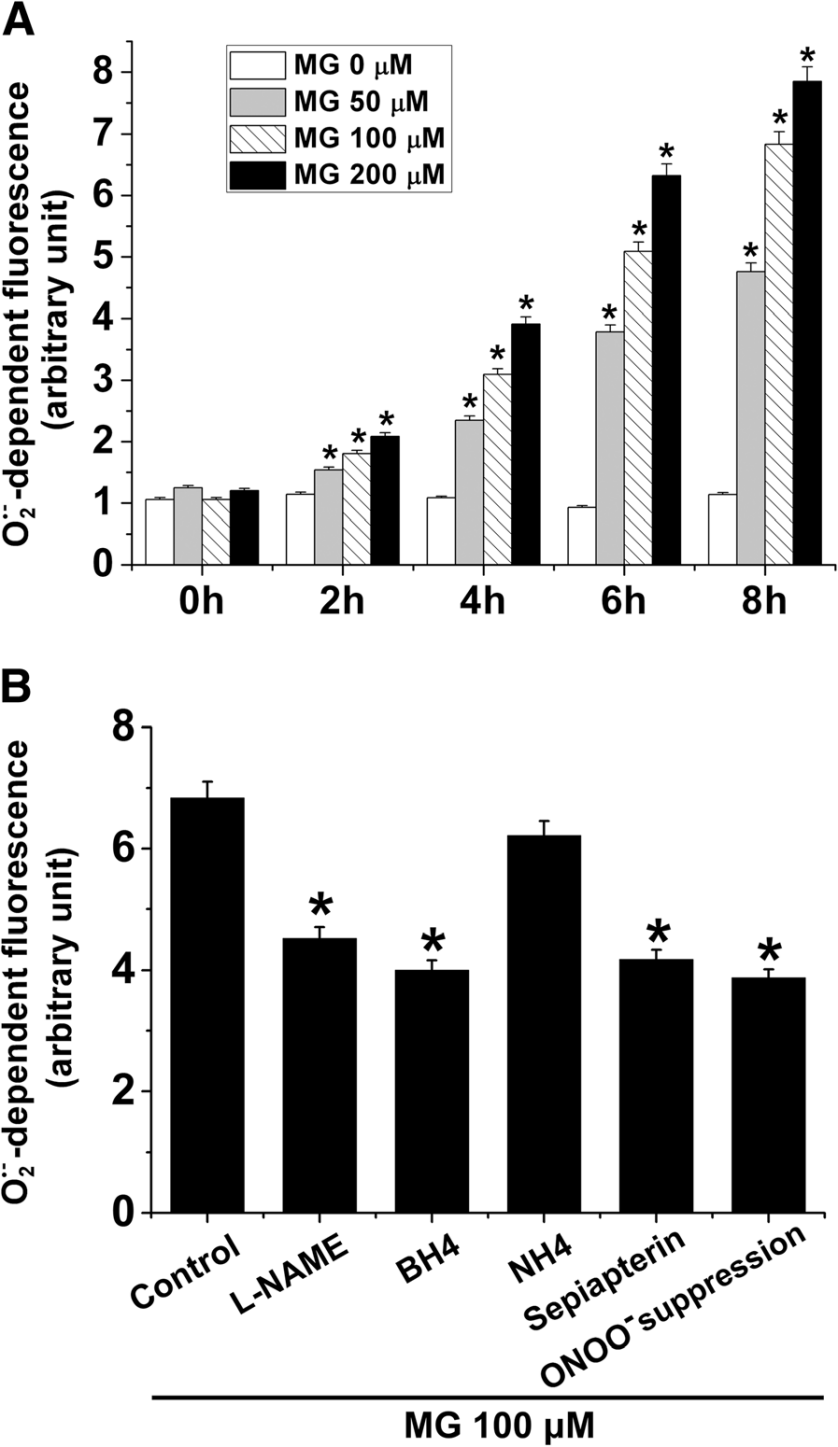
**Methylglyoxal-stimulated**$O_{2}^{•-}$**production in EA.hy926 endothelial cells. A**. $O_{2}^{•-}$ generation determined in EA.hy296 endothelial cells treated with different concentrations of MG (0, 50, 100 and 200 μM) for 0, 2, 4, 6 and 8 h respectively. Data are arithmetic means ± SEM (n = 6). * indicates significant difference (p < 0.05) from the absence of MG (ANOVA). **B**. $O_{2}^{•-}$ generation determined in EA.hy296 endothelial cells treated with MG (100 μM) for 8 h in the absence (Control) or in the presence of L-NAME (50 μM), BH4 (100 μM), NH4 (100 μM) or sepiapterin (20 μM) in the last 4 h of MG treatment or 24-h pre-treatment with 300 μM Tempol and 50 μM L-NAME (ONOO^-^ suppression) prior to the addition of MG. Data are arithmetic means ± SEM (n = 6). * indicates significant difference (p < 0.05) from Control (ANOVA).

To elucidate the redox imbalance triggered by MG treatment on vascular endothelial cells *in vitro*, we analyzed MG-triggered $O_{2}^{•-}$ generation using a fluorescence-based assay. Treatment of EA.hy926 endothelial cells with MG (50 – 200 μM) for 2, 4, 6 and 8 h respectively, significantly increased $O_{2}^{•-}$ production in a concentration- and time-dependent manner (Figure 1A). In another series of experiments, we analyzed the effect of biopterin supplementation (BH4, 100 μM; sepiapterin, 20 μM), pharmacological inhibition of NOS using L-NAME (50 μM) or suppression of ONOO^-^ formation using combined 24-h pretreatment with Tempol (300 μM) and L-NAME (50 μM) on MG-induced $O_{2}^{•-}$ production. As shown in Figure 1B, inhibition of NOS, suppression of ONOO^-^ formation, or supplementation of either BH4 or sepiapterin significantly attenuated MG-induced $O_{2}^{•-}$ production. Treatment of EA.hy926 cells with the BH4 control NH4 (100 μM), however, did not significantly alter MG-induced $O_{2}^{•-}$ production (Figure 1B). These results suggest eNOS-derived $O_{2}^{•-}$ production in MG-treated EA.hy296 endothelial cells.

**Figure 2:**
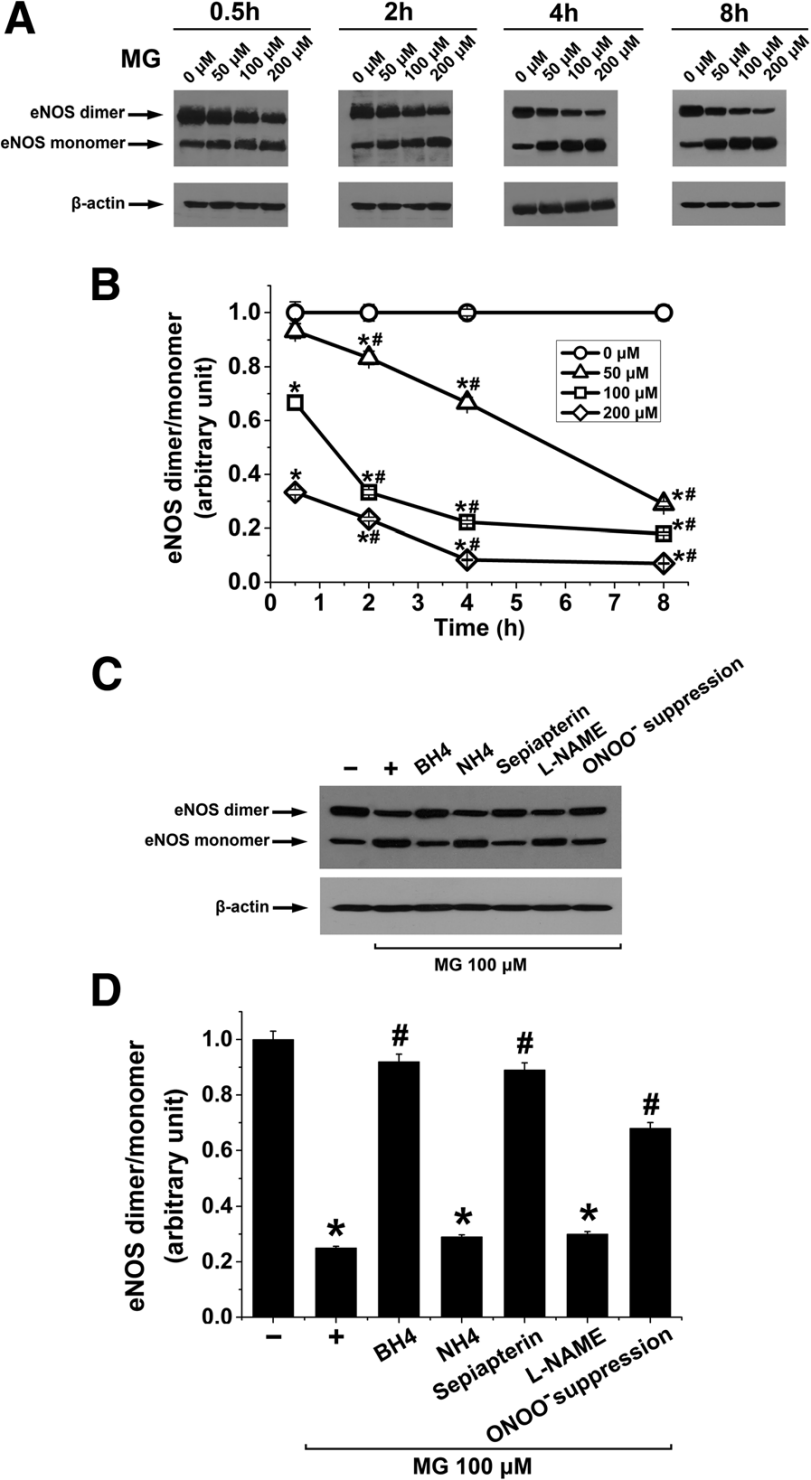
**Methylglyoxal-induced eNOS monomerization in EA.hy926 endothelial cells. A** and **B**. Original Western blots **(A)** and the respective densitometric analysis **(B)** of the relative abundance of eNOS dimers over eNOS monomers (relative to β-actin) in EA.hy296 endothelial cells treated with different concentrations of MG (0 μM, circles; 50 μM, triangles; 100 μM, squares; and 200 μM, diamonds) for 0.5, 2, 4, and 8 h respectively. Data are arithmetic means ± SEM (**A**, representative of five experiments; **B**, n = 5). * indicates significant difference (p < 0.05) from the absence of MG (ANOVA). # indicates significant difference (p < 0.05) from time 0.5 h (ANOVA). **C** and **D**. Original Western blots **(C)** and the respective densitometric analysis **(D)** of the relative abundance of eNOS dimers over eNOS monomers (relative to β-actin) in EA.hy296 endothelial cells incubated in the absence (-) or in the presence of MG (100 μM) for 8 h alone (+) or with 100 μM BH4, 100 μM NH4, 20 μM sepiapterin or 50 μM L-NAME in the last 4 h of MG treatment or with 24-h pre-treatment with 300 μM Tempol and 50 μM L-NAME (ONOO^-^ suppression). Data are arithmetic means ± SEM (**C**, representative of four experiments; **D**, n = 4). * indicates significant difference (p < 0.05) from the absence of MG (ANOVA). # indicates significant difference (p < 0.05) from MG treatment alone (ANOVA).

Next, we explored the effect of MG on eNOS monomerization in EA.hy926 endothelial cells. Treatment of EA.hy926 cells with MG (50 – 200 μM) for 0.5, 2, 4, and 8 h respectively, significantly decreased eNOS dimer/monomer ratio in a concentration- and time-dependent manner (Figure 2A and 2B). These results, together with the effect of L-NAME inhibition on MG-induced $O_{2}^{•-}$ production (Figure 1B), suggest that MG treatment induces eNOS uncoupling in EA.hy926 cells. We further tested the effect of biopterin supplementation, L-NAME addition and suppression of ONOO^-^ formation on MG-elicited eNOS monomerization. As shown in Figure 2C and 2D, treatment of EA.hy926 cells with MG (100 μM) for 8 h significantly reduced eNOS dimer/monomer ratio, an effect that was significantly blunted by the administration of BH4 (100 μM, 4 h), sepiapterin (20 μM, 4 h) or by ONOO^-^ suppression (300 μM Tempol and 50 μM L-NAME, 24 h). Treatment with L-NAME (50 μM, 4 h) alone or the BH4 control NH4 (100 μM, 4 h) did not modify eNOS dimer/monomer ratio (Figure 2C and 2D).

**Figure 3:**
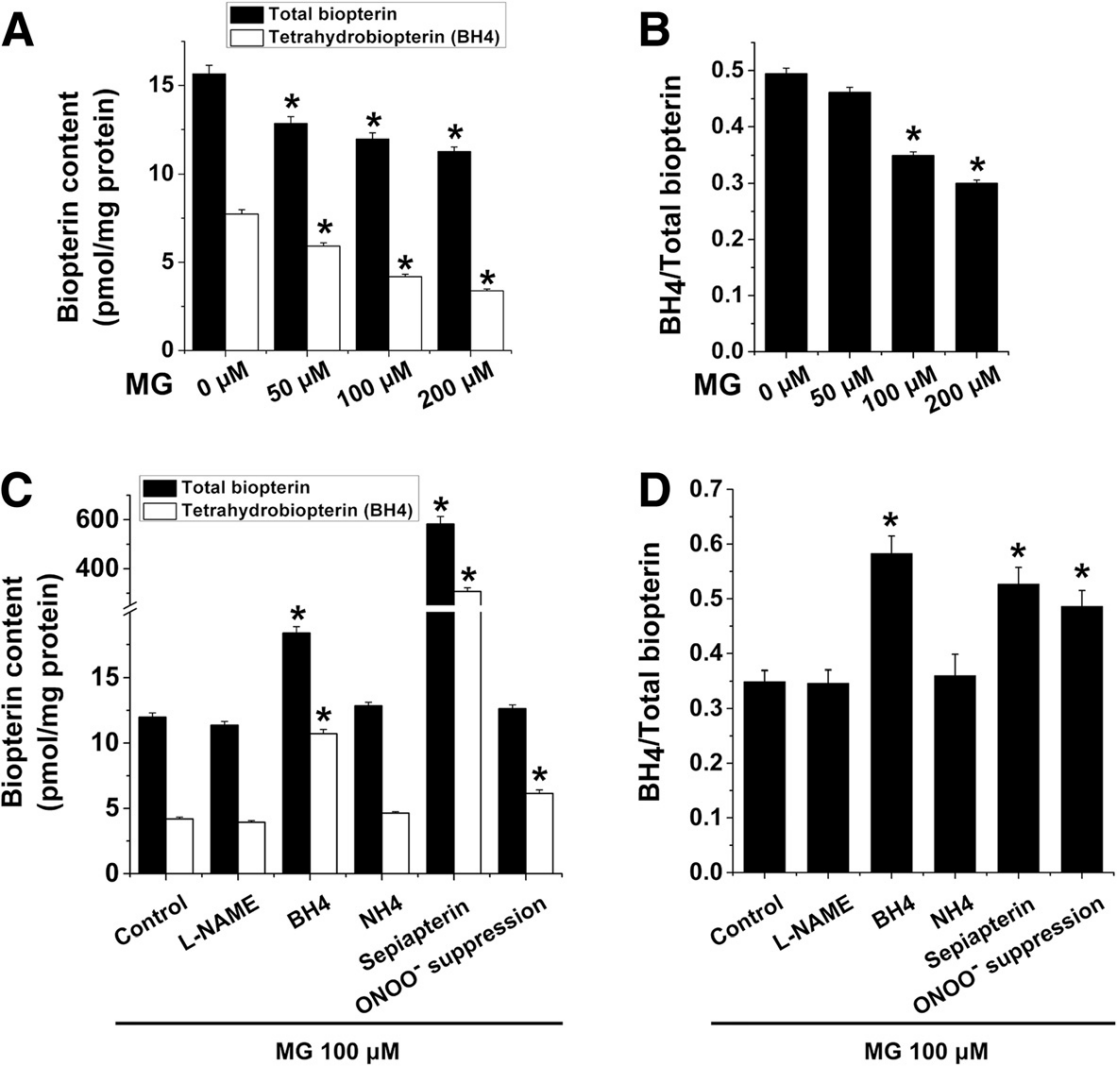
**Effect of methylglyoxal on cellular biopterin levels in EA**.**hy926 endothelial cells. A** and **B**. Total cellular biopterin (pmol/mg protein; black bars; **A)** and BH4 (pmol/mg protein; white bars; **A)** and the respective BH4/total biopterin ratio **(B)** determined in EA.hy296 endothelial cells treated with different concentrations of MG (0, 50, 100 and 200 μM) for 8 h. Data are arithmetic means ± SEM (n = 5). * indicates significant difference (p < 0.05) from the absence of MG (ANOVA). **C** and **D**. Total cellular biopterin (pmol/mg protein; black bars; **C)** and BH4 (pmol/mg protein; white bars; **C)** and the respective BH4/total biopterin ratio **(D)** determined in EA.hy296 endothelial cells treated with MG (100 μM) for 8 h in the absence (Control) or in the presence of L-NAME (50 μM), BH4 (100 μM), NH4 (100 μM) or sepiapterin (20 μM) in the last 4 h of MG treatment or with 24-h pre-treatment with 300 μM Tempol and 50 μM L-NAME (ONOO^-^ suppression). Data are arithmetic means ± SEM (n = 5). * indicates significant difference (p < 0.05) from Control (ANOVA).

To further elucidate MG-induced eNOS uncoupling, we determined the cellular BH4 levels in EA.hy926 cells following MG treatment. As depicted in Figure 3A, treatment of EA.hy926 cells with MG (50 – 200 μM) for 8 h significantly decreased total cellular biopterin and BH4 levels in a concentration-dependent manner. More importantly, treatment of EA.hy926 cells resulted in a reduction of BH4/total biopterin ratio, an effect reaching statistical significance at 100 μM MG concentration (Figure 3B). We then explored the effect of biopterin supplementation, L-NAME addition and suppression of ONOO^-^ formation on MG-induced reduction in cellular BH4 levels. As illustrated in Figure 3C, 4-h supplementation of BH4 (100 μM) or sepiapterin (20 μM) in MG-treated EA.hy926 cells significantly increased total cellular biopterin and BH4 levels suggesting that exogenous biopterin repletion dissipated MG-induced reduction in cellular BH4 contents associated with eNOS uncoupling. Suppression of ONOO^-^, however, did not significantly modify total cellular biopterin levels but significantly increased cellular BH4 levels in MG-treated cells (Figure 3C). Neither the BH4 control NH4 (100 μM) nor L-NAME (50 μM) alone significantly altered cellular BH4 and total biopterin levels (Figure 3C). Similarly, MG-induced reduction of cellular BH4/total biopterin ratio was significantly attenuated by supplementation with BH4 or sepiapterin or by ONOO^-^ suppression but not by the BH4 control NH4 or by L-NAME alone (Figure 3D).

**Figure 4:**
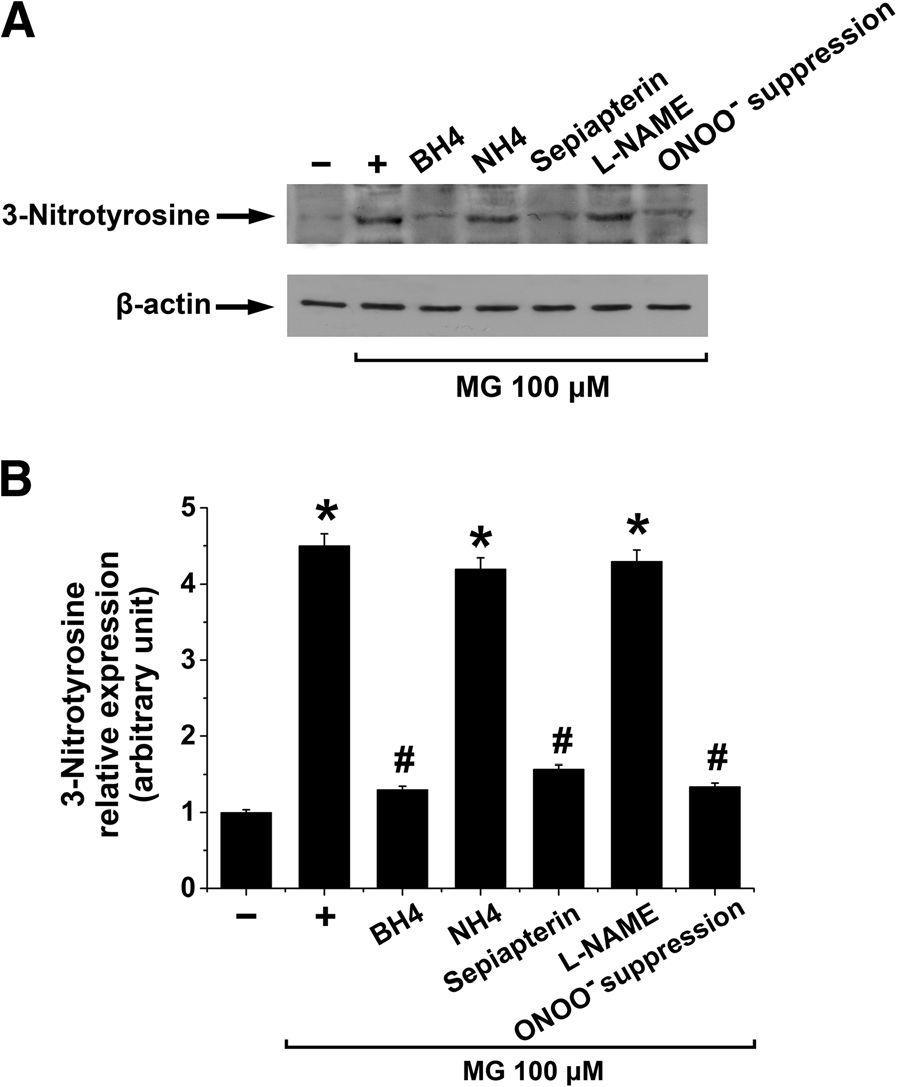
**Effect of methylglyoxal on tyrosine nitration in EA.hy926 endothelial cells. A** and **B**. Original Western blots **(A)** and the respective densitometric analysis **(B)** of the relative abundance of 3-nitrotyrosine (relative to β-actin) in EA.hy296 endothelial cells incubated in the absence (-) or in the presence of MG (100 μM) for 8 h alone (+) or with 100 μM BH4, 100 μM NH4, 20 μM sepiapterin, or 50 μM L-NAME in the last 4 h of MG treatment or with 24-h pre-treatment with 300 μM Tempol and 50 μM L-NAME (ONOO^-^ suppression). Data are arithmetic means ± SEM (**A**, representative of three experiments; **B**, n = 3). * indicates significant difference (p < 0.05) from the absence of MG (ANOVA). # indicates significant difference (p < 0.05) from MG treatment alone (ANOVA).

Uncoupling of eNOS is associated with enhanced cellular nitration of tyrosine [17]. In a further series of experiments we examined the abundance of 3NT in EA.hy926 cells treated with MG and the effect of biopterins, L-NAME or ONOO^-^ suppression after MG. As shown in Figure 4, treatment of EA.hy926 cells with 100 μM of MG for 8 h significantly increased the expression of 3NT, an effect that was significantly blunted by BH4 (100 μM, 4 h), sepiapterin (20 μM, 4 h) or ONOO^-^ suppression (300 μM Tempol and 50 μM L-NAME, 24 h) but not by the BH4 control NH4 (100 μM, 4 h) or L-NAME (50 μM, 4 h) suggesting that biopterin-sensitive eNOS uncoupling was triggered by MG (Figure 4A and 4B). The effect of ONOO^-^ suppression on the reduction of 3NT expression by the combination of Tempol (300 μM) and L-NAME (50 μM) was higher than the administration of Tempol (300 μM) alone (data not shown).

**Figure 5:**
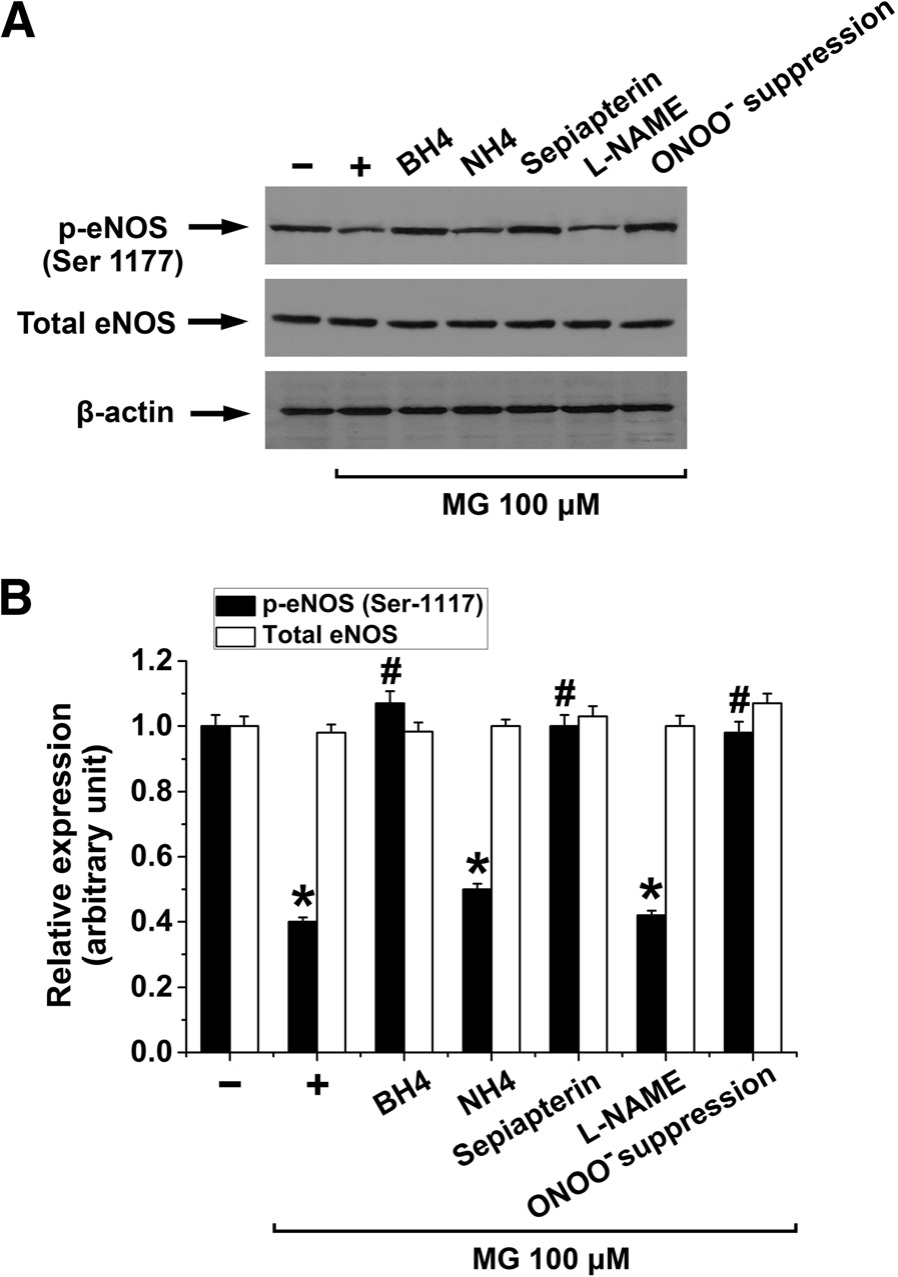
**Effect of methylglyoxal on eNOS phosphorylation in EA.hy926 endothelial cells. A** and **B**. Original Western blots **(A)** and the respective densitometric analysis **(B)** of the relative abundance of total eNOS protein (white bars; relative to β-actin) and Ser-1177 phospho-eNOS (black bars; relative to β-actin) in EA.hy296 endothelial cells incubated in the absence (-) or in the presence of MG (100 μM) for 8 h alone (+) or with 100 μM BH4, 100 μM NH4, 20 μM sepiapterin or 50 μM L-NAME in the last 4 h of MG treatment or with 24 h pre-treatment with 300 μM Tempol and 50 μM L-NAME (ONOO^-^ suppression). Data are arithmetic means ± SEM (**A**, representative of three experiments; **B**, n = 3). * indicates significant difference (p < 0.05) from the absence of MG (ANOVA). # indicates significant difference (p < 0.05) from MG treatment alone (ANOVA).

To further unravel the modulation of eNOS functions by MG, we investigated the effect of biopterin supplementation, ONOO^-^ suppression and NOS inhibition on eNOS phosphorylation (Ser-1177) associated with eNOS uncoupling. To this end, treatment of EA.hy926 cells with 100 μM of MG for 8 h did not modify total eNOS but significantly decreased phosphorylation of eNOS (Ser-1177), an effect that was significantly blunted by BH4 (100 μM), sepiapterin (20 μM) or ONOO^-^ suppression but not by the BH4 control NH4 (100 μM) or L-NAME (50 μM) suggesting that both eNOS phosphorylation (Ser-1177) and eNOS uncoupling are closely linked to MG-triggered endothelial dysfunction (Figure 5A and 5B).

## Discussion

In this study we provide conclusive biological evidence that treatment of EA.hy926 endothelial cells with MG triggers eNOS hypophosphorylation and eNOS uncoupling *in vitro*. We show that MG treatment enhances $O_{2}^{•-}$ generation and 3NT expression and decreases Ser-1177 phosphorylated eNOS, eNOS dimer/monomer ratio and cellular levels of BH4 and BH4/total biopterin ratio in endothelial cells. Moreover, we demonstrate that pharmacological suppression of eNOS uncoupling by L-NAME, exogenous BH4 or sepiapterin administration, and ONOO^-^ suppression counteracted MG-induced eNOS uncoupling, 3NT upregulation and eNOS hypophosphorylation.

Oxidative stress is a salient feature of the pathophysiology of diabetes. The glycolysis metabolite MG was previously shown to affect redox balance by stimulating ROS production and modulating the expression and functions of cytoprotective molecules such as glutathione [40], superoxide dismutase [41], glyoxalase [42] and H_2_S [43]. Increased NADPH oxidase-derived $O_{2}^{•-}$ reacts with eNOS-derived NO to generate ONOO^-^ which oxidizes BH4 to BH2. BH4 reduction has been implicated in the pathogenesis of a variety of conditions such as hyperphenylalaninemia [44], diabetes [45], ischemia-reperfusion injury [46], hypertension [47], Alzheimer disease and Parkinson’s disease [48], and many of these pathological conditions were shown to be ameliorated by exogenous BH4 [44-47] or sepiapterin [49-51] supplementation. BH4 is critical for the maintenance of eNOS dimers and is functionally related to S-glutathionylation, a powerful regulator of eNOS uncoupling [52]. Our data that MG-triggered $O_{2}^{•-}$ generation was inhibitable by L-NAME addition, BH4 or sepiapterin supplementation and ONOO^-^ suppression clearly suggest the presence and contribution of uncoupled eNOS to $O_{2}^{•-}$ generation in endothelial cell functions triggered by MG.

Mounting evidence suggests that phosphorylation of the residue Ser-1177 of eNOS via AKT stimulates NO production [53]. The effects of eNOS phosphorylation on eNOS uncoupling and $O_{2}^{•-}$ production are not completely understood. Ser-1177 phosphorylation was shown to inhibit the Ca^2+^ sensitivity of eNOS and thus fosters Ca^2+^-independent $O_{2}^{•-}$ generation [54]. In this study we tested the effect of BH4 supplementation on the hitherto known reduction of eNOS phosphorylation triggered by MG [30]. Our data show that supplementation of either BH4 or sepiapterin reversed MG-triggered reduction in Ser-1177 eNOS phosphorylation and enhanced eNOS uncoupling. Similar to BH4, other pharmacological agents such as nifedipine [55], nicorandil [56], and telmisartan [57] were shown to inhibit eNOS uncoupling and enhance eNOS phosphorylation and may, therefore, be beneficial in diabetic endothelial dysfunction. Furthermore, endogenous mediators such as C-reactive protein [58], IGF-1 [59], calcitriol [60], and 20-hydroxyeicosatetraenoic acid [61] were reported to modulate eNOS-associated functions. In this study we demonstrate for the first time the eNOS monomerization induced by the glycolysis metabolite MG and the effects of BH4 and sepiapterin supplementation on MG-modulated eNOS-associated functions in endothelial cells.

Uncoupling of eNOS is characterized by augmented tyrosine nitration which strongly indicates ONOO^-^-triggered cellular injury in diabetic hyperglycemia [62]. Increased placental protein tyrosine nitration was documented in diabetic patients [63] and, in animal models of diabetes, 3NT was shown to be enhanced in the kidney [64] and in the ventricle and lens [65]. Similarly, 3NT is upregulated in diabetic cardiomyopathy [66]. Our finding that BH4 but not NH4 supplementation abrogated enhanced 3NT expression in MG-treated endothelial cells supports the view that MG-induced depletion of eNOS factor BH4 is critical for MG-triggered eNOS uncoupling and endothelial dysfunction. Similar observations were also made in the diabetic heart subjected to ischemia-reperfusion injury [46], clearly indicating that exogenous BH4 supplementation may ameliorate diabetes-associated multi-organ pathologies.

Conflicting reports on eNOS expression in diabetic endothelial dysfunction suggest the complexity of the signalling mechanisms associated with eNOS during hyperglycemia. Treatment of endothelial cells with high concentrations of glucose was reported to have decreased eNOS protein expression *in vitro*[67]. It was also reported that the abundance of total and phosphorylated eNOS was reduced in internal mammary arteries of diabetic patients [68]. In old Zucker diabetic fatty rats, eNOS protein expression was demonstrated to be decreased [69]. On the contrary, in diabetic and apolipoprotein E-deficient mice, eNOS mRNA levels were augmented [70]. In a contrasting report, however, eNOS expression was shown to be unaltered in type 2 diabetic db/db mice [71]. Along those lines, the discordant effects of MG treatment on eNOS functions reported previously may, thus, not be unexpected [27-30]. It is, however, intriguing to speculate that MG may be one of the essential metabolites that triggers eNOS hypophosphorylation and uncoupling encountered in diabetes.

## Conclusions

We conclude that the intermediate glycolysis metabolite MG is a powerful modulator of eNOS functions in vascular endothelial cells *in vitro*. Administration of BH4 or its precursor sepiapterin or suppression of ONOO^-^ formation replenishes cellular BH4 levels, reverses hypophosphorylation of eNOS and ameliorates $O_{2}^{•-}$ generation, 3NT upregulation and eNOS monomerization that are associated with MG-triggered eNOS uncoupling in endothelial cells. These pharmacological measures may indicate promising therapeutic benefits in MG-sensitive endothelial dysfunction in diabetes.

## Abbreviations

MG: Methylglyoxal; eNOS: Endothelial nitric oxide synthase; NO: Nitric oxide; BH4: 5,6,7,8-tetrahydrobiopterin; NH4: 5,6,7,8-tetrahydroneopterin; BH2: 7,8-dihydrobiopterin; L-NAME: N^G^-nitro-L-arginine methyl ester; Tempol: 1-oxyl-2,2,6,6-tetramethyl-4-hydroxypiperidine; $O_{2}^{•-}$: Superoxide; ONOO-: Peroxynitrite; 3NT: 3-Nitrotyrosine; NADPH: Nicotinamide adenine dinucleotide phosphate; HSP90: Heat shock protein 90; LOX-1: Lectin-type oxidized LDL receptor 1; IGF-1: Insulin-like growth factor 1.

## Competing interests

The authors declare that they have no conflict of interest.

## Authors’ contributions

Participated in research design: YS, SMQ, LW, and LL; conducted experiments: YS; performed data analysis and interpretation: YS, SMQ, and LL; contributed to the writing of the manuscript: YS, SMQ, and LL; critically revised the manuscript: LW and LL; all authors read and approved the final manuscript.
